# Mutation profiling in differential diagnosis between TdT‐positive high‐grade/large B‐cell lymphoma and B‐lymphoblastic leukaemia/lymphoma

**DOI:** 10.1002/path.6476

**Published:** 2025-10-06

**Authors:** Maria‐Myrsini Tzioni, Francesco Cucco, Lívia Rásó‐Barnett, Zi Chen, Andrew Wotherspoon, Katrin S Kurz, Ewelina Madej, Jasmine Makker, Anna E Strazda, Fang Guo, Caoimhe Egan, Elizabeth Soilleux, Liz Hook, Laszlo Krenacs, Julia T Geyer, Camille Laurent, Luc Xerri, Lenaïg Mescam, Lukas Plank, Lise Mette Rahbek Gjerdrum, Nicolas Lopez‐Hisijos, Timothy Greiner, Joseph Khoury, Wolfram Klapper, Ilske Oschlies, Andreas Rosenwald, German Ott, Ming‐Qing Du

**Affiliations:** ^1^ Department of Pathology University of Cambridge Cambridge UK; ^2^ Institute of Clinical Physiology, CNR Pisa Italy; ^3^ The Haematopathology and Oncology Diagnostic Service Cambridge University Hospitals NHS Foundation Trust Cambridge UK; ^4^ Department of Histopathology The Royal Marsden Hospital London UK; ^5^ Department of Clinical Pathology Robert‐Bosch‐Krankenhaus, and Dr. Margarete Fischer‐Bosch Institute for Clinical Pharmacology Stuttgart Germany; ^6^ Hubei Cancer Hospital, Tongji Medical College Huazhong University of Science and Technology Wuhan Hubei PR China; ^7^ Laboratory of Tumor Pathology and Molecular Diagnostics Szeged Hungary; ^8^ Department of Pathology and Laboratory Medicine Weill Cornell Medical College New York NY USA; ^9^ Department of Bio‐Pathology Institut Universitaire Cancer‐Oncopole, Centre de Recherches en Cancérologie de Toulouse INSERM U1037 Toulouse France; ^10^ Department of Pathology, Institut Paoli‐Calmettes, Centre de Recherche en Cancérologie de Marseille Aix‐Marseille University Marseille France; ^11^ Department of Pathological Anatomy University Hospital Martin, Jessenius Faculty of Medicine, Comenius University Martin Slovakia; ^12^ Department of Pathology Copenhagen University Hospital–Zealand University Hospital Roskilde Denmark; ^13^ Department of Pathology, Microbiology, and Immunology Vanderbilt University Medical Center Nashville TN USA; ^14^ Department of Pathology, Microbiology, and Immunology University of Nebraska Medical Center Omaha NE USA; ^15^ Sektion Hämatopathologie und Lymphknotenregister Universitätsklinikum Schleswig‐Holstein, Campus Kiel Kiel Germany; ^16^ Institute of Pathology University of Würzburg Würzburg Germany

**Keywords:** TdT expression, high‐grade B‐cell lymphoma, B‐lymphoblastic leukaemia/lymphoma, somatic hypermutation, differential diagnosis

## Abstract

Terminal deoxynucleotidyl transferase (TdT) is occasionally expressed in large B‐cell lymphoma (LBCL), and this causes difficulty in differential diagnosis from B‐lymphoblastic leukaemia/lymphoma (B‐ALL/LBL). We reviewed 31 cases of TdT‐positive LBCL and B‐ALL/LBL, and their final diagnosis included 19 diffuse large/high‐grade BCLs with *MYC* and *BCL2* rearrangements (five DLBCL‐*MYC*/*BCL2*, 14 HGBCL‐*MYC/BCL2*), three DLBCL not otherwise specified (NOS), three HGBCL‐NOS, four B‐ALL/LBL, and two unclassifiable cases. TdT was variably expressed in all these cases, without any clear demarcation among different groups. Loss or partial loss of CD20 expression was seen in 13/17 DLBCL/HGBCL‐*MYC*/*BCL2*, 2/3 HGBCL‐NOS, and 2/2 unclassified, albeit not in DLBCL‐NOS. Expression of BCL6 and/or MUM1 was seen in 3/4 B‐ALL/LBLs and 2/2 unclassified. Next‐generation sequencing revealed characteristic mutations associated with follicular lymphoma and its high‐grade transformation in each DLBCL/HGBCL‐*MYC*/*BCL2*, and also frequent variants in genes targeted by somatic hypermutation (SHM) in almost all DLBCL/HGBCL‐*MYC*/*BCL2*, DLBCL‐NOS, and HGBCL‐NOS but one case. In contrast, such mutations were absent in B‐ALL/LBL. There were no pathognomonic mutations in the two unclassifiable cases, although one showed a moderate level of somatic mutations in its rearranged *IGHV*. Furthermore, in three cases of TdT‐positive HGBCL‐*MYC*/*BCL2*, studies of previous or concurrent follicular lymphoma demonstrated their divergent evolution from an *IGH*::*BCL2*‐positive cell population following acquisition of *MYC* translocation. In conclusion, mutation profiling analysis including the SHM target genes is highly valuable in the differential diagnosis between TdT‐positive LBCL and B‐ALL/LBL. © 2025 The Author(s). *The Journal of Pathology* published by John Wiley & Sons Ltd on behalf of The Pathological Society of Great Britain and Ireland.

## Introduction

Immunophenotyping is pivotal in the diagnosis and classification of haematolymphoid malignancies. By comparing the immunophenotype of malignant cells with those of normal lymphoid cells at various stages of their development and maturation, it is possible to determine the tumour cell‐of‐origin, hence enabling its diagnosis. However, some of the immunophenotypic markers may be expressed aberrantly in lymphoma cells, and this may cause challenges in their application to lymphoma diagnosis. One such immunophenotypic marker is terminal deoxynucleotidyl transferase (TdT).

TdT is typically expressed in pro‐ and pre‐B cells in the bone marrow but is downregulated and not expressed in mature B cells in peripheral lymphoid tissues. Thus, TdT is regarded as a marker of precursor B cells and their derived leukaemias and lymphomas, i.e. B‐lymphoblastic leukaemias/lymphomas (B‐ALLs/LBLs). However, TdT is also expressed in rare cases of diffuse large B‐cell lymphoma (DLBCL) transformed from follicular lymphoma (FL) with dual *MYC* and *BCL2* rearrangements [1]. This had been used to argue for the classification of rare cases of TdT‐positive blastoid B‐cell lymphoma with both *MYC* and *BCL2* translocations as B‐LBL in the revised fourth edition of the World Health Organization classification of haematolymphoid tumours (WHO‐HAEM4R) [2]. However, these cases are CD34‐negative, often bear a mature B‐cell phenotype, and show a mutation profile similar to those of transformed FL [3, 4, 5, 6]. These findings together with their exclusive occurrence in adults support their origin from mature B cells with TdT expression being aberrant. Accordingly, these cases are now designated as a subtype of diffuse large/high‐grade B‐cell lymphoma with *MYC*/*BCL2* rearrangement (DLBCL/HGBCL‐*MYC*/*BCL2*, or DLBCL/HGBCL‐*MYC*/*BCL2*/*BCL6* when an additional *BCL6* rearrangement is present) in the fifth edition of the WHO classification of haematolymphoid tumours (WHO‐HAEM5) [7].

In general, DLBCL/HGBCL‐*MYC*/*BCL2* can be readily diagnosed by combining histopathological/immunophenotypic assessment with interphase FISH analysis for these translocations. In addition, DLBCL/HGBCL‐*MYC*/*BCL2* bears characteristic mutation signatures including pathogenic changes associated with classic FL as well as those associated with high‐grade transformation, serving as a robust genetic basis for differential diagnosis [8, 9, 10, 11, 12]. However, aberrant TdT expression may also be seen in DLBCL/HGBCL without *MYC* and *BCL2* translocation [1], and such cases may also bear features of immaturity such as loss of CD20, BCL6, and/or immunoglobulin light chain expression, posing a real challenge in their differential diagnosis from B‐ALL/LBL due to a lack of pathognomonic genetic changes in the former [13]. On the other hand, B‐ALLs/LBLs, including rare cases with *MYC* and *MYC/BCL2* translocations, occur commonly in children [13] but are also seen in young adults, thus overlapping with the age range of patients with DLBCL/HGBCL [14, 15, 16].

Given the distinct cell of origin between TdT‐positive DLBCL/HGBCL and B‐ALL/LBL and potential differences in their mutation profiles, we investigated somatic mutations in both rearranged *IGHV* genes and lymphoma genes (*n* = 187) by targeted next‐generation sequencing (NGS) and performed integrated analysis to explore their utility in differential diagnosis. We also investigated the evolutionary history of TdT‐positive transformed FL in cases where biopsies from paired metachronous lymphomas were available.

## Materials and methods

### Ethical approval and case material

The study was performed in accordance with local ethical guidelines for the research use of tissue materials with the approval of the ethics committees of the involved institutions (05‐Q1604‐10). A total of 31 cases of TdT‐positive aggressive B‐cell lymphoma were retrieved from the authors' institutions, and their histological diagnosis was reviewed, with a final diagnosis assigned according to WHO‐HAEM5 [7].

### Interphase fluorescence *in situ* hybridisation (FISH)

*MYC*, *BCL2*, and *BCL6* translocation status was available in the majority of cases from routine haematopathological diagnosis. Any missing translocation data were completed retrospectively together with further interphase FISH with *MYC*/*IGH* (Abbott Laboratories, Maidenhead, UK), *MYC*/*IGK*, and *MYC*/*IGL* (Cytocell, Cambridge, UK) dual fusion probes.

### Immunohistochemistry

The majority of the immunophenotypic data were available from routine haematopathological diagnosis, and any missing data on MYC and CD34 were collected retrospectively under the same conditions as routine histological diagnosis (supplementary material, Table S1).

Additional TdT immunocytochemistry was performed on formalin‐fixed paraffin‐embedded cell clots of DLBCL cell lines (both *MYC* and *BCL2* translocation‐positive: OCI‐Ly4, OCI‐LY18, Su‐DHL6, SC1, DB; *MYC* translocation‐positive: RIVA; *MYC* translocation‐negative: OCI‐Ly3, OCI‐Ly10, Karpas‐422, Su‐DHL2) and the Burkitt lymphoma cell line BJAB.

### DNA extraction and quality assessment

Histology was reviewed and the areas containing confluent lymphoma cells (>40%) in each tissue specimen were microdissected on consecutive tissue sections. DNA was extracted using the QIAamp DNA Micro Kit (QIAGEN, Crawley, UK), quantified with a Qubit® Fluorometer (Life Technologies, Carlsbad, CA, USA), and assessed for quality by PCR [17].

### Mutation analysis by targeted next‐generation sequencing

This was carried out using a customised panel of 187 genes that are recurrently mutated in FL and DLBCL/HGBCL (supplementary material, Table S2). Apart from TdT‐positive DLBCL/HGBCL, four cases of TdT‐positive B‐ALL/LBL were included for comparative analysis to depict their difference in mutation pattern among the panel genes investigated. A total of 80–200 ng of FFPE tissue‐derived DNA was fragmented using the Covaris E220 Focused Ultrasonicator (Covaris, Brighton, UK). For each DNA sample, an indexed library was prepared with the xGen™ UDI‐UMI indexes [Integrated DNA Technologies, Inc. (IDT), Coralville, IA, USA] using the TWIST protocol and then pooled for target enrichment using the TWIST probes (TWIST Biosciences, South San Francisco, CA, USA). The enriched DNA targets were amplified by PCR and pooled libraries were sequenced using the Illumina NextSeq 2000 platform (Illumina, San Diego, CA, USA) (2 × 100 bp paired‐end sequencing protocol). The sequence data analysis, variant calling, and filtering were performed as described in our previous studies [8, 17, 18].

For DNA samples with sub‐optimal quality (PCR amplification of genomic fragments ≤ 300 bp), targeted sequencing was performed in duplicates and only variants detected in both replicates were considered as a true alteration (supplementary material, Figure S1).

### Clonality analysis of the rearranged immunoglobulin heavy chain (IGH) genes

This was performed by adopting the BIOMED‐2 assays, followed by NGS using the 2 × 250 bp paired‐end sequencing protocol (Illumina NovaSeq X sequencer) (Supplementary materials and methods and supplementary material, Table S3).

### Statistical analyses

Comparison of the mutation load among different groups was assessed using a Wilcoxon rank‐sum test with two‐sided *p* values.

## Results

### Histopathological features and their value in differential diagnosis

**Figure 1:**
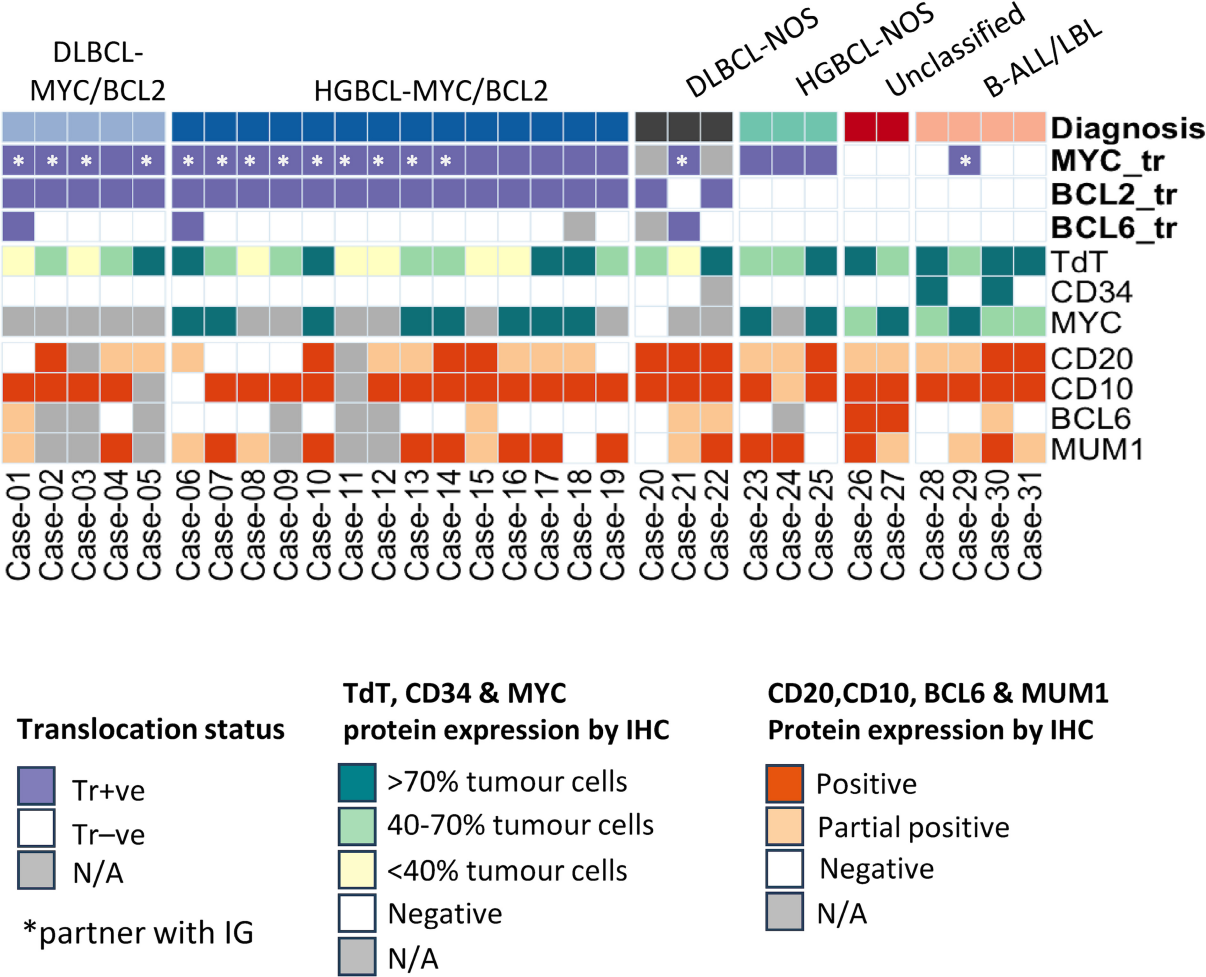
Summary of chromosomal translocations and key immunophenotype. TdT, CD34, and MYC immunohistochemistry was centrally performed or reviewed in the Cambridge laboratory and their expression level is semi‐quantified, while CD20, CD10, BCL6, and MUM1 immunostaining results are from a referral laboratory and recorded in a non‐quantitative manner. Except for exclusive CD34 expression in two cases of B‐ALL/LBL, there is no clear demarcation among the expression of other immunophenotypic markers between different groups. B‐ALL/LBL, B‐lymphoblastic leukaemia/lymphoma; DLBCL, diffuse large B‐cell lymphoma; HGBCL, high‐grade B‐cell lymphoma; Tr +ve, translocation‐positive; Tr −ve, translocation‐negative; IHC, immunohistochemistry; N/A, not available.

For each case, the histological diagnosis was reviewed and where possible a final diagnosis was made according to WHO‐HAEM5. The final diagnosis included 19 diffuse large/high‐grade BCLs with *MYC* and *BCL2* rearrangements (five with DLBCL morphology including one with an additional *BCL6* rearrangement and 14 with HGBCL morphology including one with an additional *BCL6* rearrangement), three DLBCL‐NOS, three HGBCL‐NOS, four B‐ALL/LBLs, and two cases unclassifiable due to equal evidence for both an immature (strong TdT expression with partial loss of both CD20 and CD79a expression) and a mature (expression of both BCL6 and MUM1 together with no CD34 expression) immunophenotype (Table 1 and Figure 1).

**Table 1:** Summary of the clinical and laboratory results of Cases 26–31 investigated.

	Case‐26	Case‐27	Case‐28	Case‐29	Case‐30	Case‐31
Diagnosis	Unclassified	Unclassified	B‐ALL/LBL	B‐ALL/LBL	B‐ALL/LBL	B‐ALL/LBL
Age (years)/sex	70/F	74/M	63/F	4/F	80/F	75/M
Clinical history	Pancytopenia	B symptoms, splenomegaly, anaemia, right thyroid nodule with histopathology consistent with B‐ALL/LBL		Pancytopenia	Thrombocytopenia and leukoerythroblastic picture	Pancytopenia and lytic bone lesion
Nodal and splenic involvement	N/A	Splenomegaly, no lymphadenopathy by CT	Stage IV, wide lymphadenopathy plus pancreas, nasopharynx, uterine, and peritoneal lesions	Splenomegaly, no lymphadenopathy by CT	N/A	N/A
Biopsy site	Bone marrow	Bone marrow	Lymph node	Bone marrow	Bone marrow	Bone marrow
Histopathology	Diffuse infiltration by lymphoblastoid cells	Diffuse infiltration by lymphoblastoid cells	Diffuse infiltration by lymphoblastoid cells	Diffuse infiltration by lymphoblastoid cells	Diffuse infiltration by large atypical lymphocytes with moderate pleomorphism	Diffuse infiltration by lymphoblastoid cells
Immunophenotype						
PAX5	Diffuse positive	Diffuse positive	N/A	N/A	Diffuse positive	N/A
CD20	Positive in ~90%	Positive in ~30%	Positive in ~50%	Weak, patchy positive	Diffuse positive	Diffuse positive
CD79a	Diffuse positive with variable intensity	Diffuse positive	Diffuse positive	Diffuse positive	Diffuse positive with variable intensity	Positive in ~30%
CD10	Diffuse positive	Diffuse positive	Diffuse positive	Diffuse positive	Diffuse positive	Diffuse positive
BCL6	Weak–moderate positive	Positive	Negative	Negative	Weak partial positive	Negative
MUM1	Strong positive	Focal positive	Negative	Positive in ~40%	Weak positive	Moderate to strong positive in ~80%
Ki67	90%	90%	60–70%	100%	60%	~90%
MYC	60%	90%	60%	80%	60%	60%
TDT	Strong positive in ~100%	Variable positivity in ~50%	Moderate positive in ~95%	Moderate to strong positivity in ~40%	Strong positive in 100%	80%
CD34	Negative	Negative	Diffuse positive	Negative	Diffuse positive	Negative
Translocation						
MYC	Negative	Negative	Negative	*IGL*::*MYC*	Negative	Negative
BCL2	Negative	Negative	Negative	Negative	Negative	Negative
BCL6	Negative	Negative	Negative	Negative	Negative	Negative
Others	N/A	No FISH evidence of *BCR*::*ABL1*, *ETV6*::*RUNX1*, *TCF3*::*PBX1*, *TCF3*::*HLF*, and *KMT2A* translocations	N/A	N/A	N/A	N/A
Mutation load	Much lower than in DLBCL/HGBCL	Much lower than in DLBCL/HGBCL	Much lower than in DLBCL/HGBCL	Much lower than in DLBCL/HGBCL	Much lower than in DLBCL/HGBCL	Much lower than in DLBCL/HGBCL
Any pathognomonic mutations	No	No	No	No	No	No
SHM‐driven mutation	No	Single synonymous change	No	No	No	No
*IGHV* mutation	N/A	95% identity to germline	86% identity to germline	No	No	N/A
Treatment and outcome	N/A	Treated with UKALL60+ protocol and showed a short period of morphological and cytogenetic remission. Then bone marrow relapse; patient was treated with 3 cycles of inotuzumab and achieved morphological and cytogenetic remission	Treated with RCHOP, RDHAC, then autografted. Two years later, lymphoma relapsed, with 15% of circulating blast‐like cells and 98% in bone marrow aspiration	Treated as Burkitt's leukaemia with chemotherapy; achieved a short period of remission, then relapsed and allografted. Further disease relapse, followed by palliative chemotherapy. Died 2 years after initial diagnosis	Patient died shortly after diagnosis	Treated with 2 cycles of R‐mini‐CVD, then transferred to hospice

*Note*: B‐ALL/LBL, B‐lymphoblastic leukaemia/lymphoma; DLBCL, diffuse large B‐cell lymphoma; HGBCL, high‐grade B‐cell lymphoma; F, female; M, male; N/A, not available.

Among the 19 cases of DLBCL/HGBCL‐*MYC*/*BCL2*, 11 patients were male and eight were female, aged between 34 and 79 years (median 63 years). TdT positivity by immunohistochemistry varied considerably irrespective of the lymphoma morphology, ranging from scattered positive cells to diffuse positivity in variable proportions of lymphoma cells (<40% in seven, 40–70% in seven, and >70% in five cases), while CD34 was negative in each of the 19 cases examined. CD10 was positive in 16 of the 17 cases where data were available. CD20 was negative in five, partially positive in eight, and diffusely positive in four cases among the 17 cases where data were available.

Among the three cases of DLBCL‐NOS, two patients were female (58 and 69 years), with one carrying *IGH*::*BCL2* and the other harbouring *IGH*::*MYC* and *BCL6* translocation, while the third patient was a male (89 years). TdT was positive in 20%–90% of the tumour cells, while CD34 was negative in the two cases examined. CD10 was positive in all three cases. None of these cases showed any loss of CD20 by immunohistochemistry.

The three cases of HGBCL‐NOS were from male patients, aged between 77 and 81 years, and each carried an isolated *MYC* translocation. TdT was positive in 50–90% of tumour cells, while CD34 was negative in all three cases. CD10 was diffusely positive in two and partially positive in the third case. Two cases showed partial loss of CD20 expression, while the third case was CD20‐positive.

The four cases of B‐ALL/LBL were from one child (4 years, female) and three adults (63–80 years, one male and two females). The paediatric case carried an *IGL*::*MYC* translocation and was TdT‐positive in 40% of the tumour cells but CD34‐negative. The three adult cases lacked *MYC* translocation; two showed diffuse positivity for both TdT and CD34, and the remaining case was TdT‐positive in ~80% of tumour cells but lacked any CD34 expression. All four cases were CD10‐positive. Two cases (the paediatric case and one adult case) showed partial staining for CD20 but diffuse positivity for CD79a, with the paediatric case expressing MUM1 in 40% of tumour cells. However, the remaining two cases displayed diffuse positivity for CD20 but variable CD79a expression (variable intensity in one case and partial expression in the other), and also expressed MUM1 in the majority of tumour cells.

**Figure 2:**
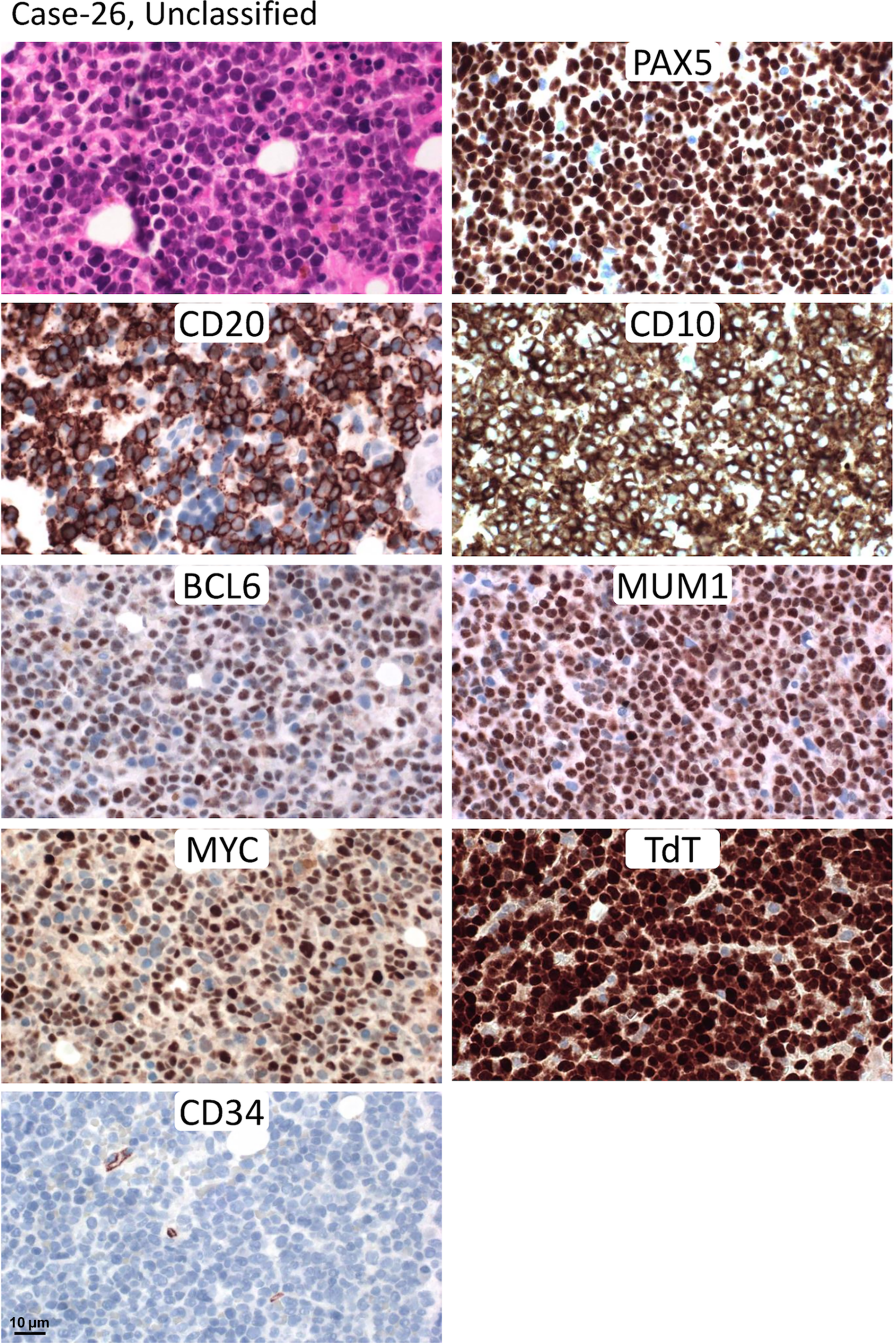
Histological and immunophenotypic presentation of Case‐26 with differential diagnosis between HGBCL‐NOS and B‐ALL/LBL. The bone marrow trephine biopsy shows infiltration by sheets of lymphoblastoid tumour cells that display diffuse PAX5 expression, but CD20 expression in ~90% tumour cells. The lymphoma cells are diffusely positive for CD10, BCL6, MUM1, MYC, and TdT, but negative for CD34.

Lastly, in two cases, the final diagnosis remained as unclassifiable between B‐ALL/LBL and HGBCL‐NOS despite careful histological review, due to equal evidence for both immature and mature immunophenotypic features. One patient was a 74‐year‐old male and the other a 70‐year‐old female. Neither of them carried a *MYC*, *BCL2*, or *BCL6* translocation. One case showed diffuse TdT positivity (Figure 2), while the other displayed TdT expression in 50% of tumour cells (supplementary material, Figure S2). Both were CD34‐negative and CD10‐positive. One case showed variable staining of CD20 and CD79a in 90% of tumour cells, and was positive for BCL6 (weak) and MUM1 (strong) (Figure 2). The other case displayed partial expression of CD20 (~30%) and CD79a (90%, variable intensity) and diffuse positivity for BCL6, and focal positivity for MUM1 (supplementary material, Figure S2).

### Genetic features and their value in differential diagnosis

**Figure 3:**
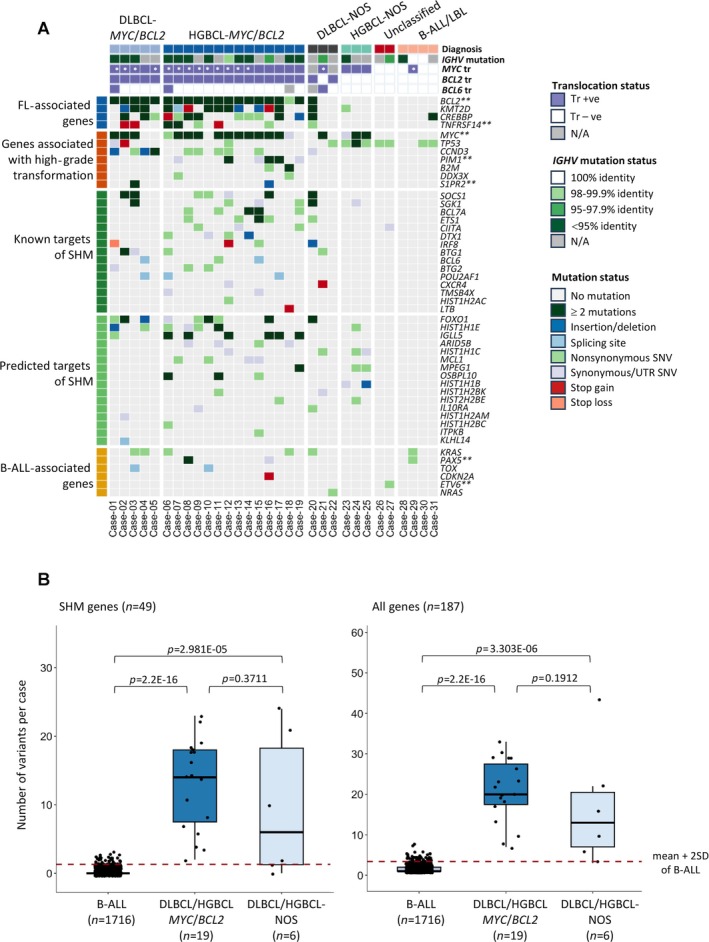
Comparison of the mutation profiles among different groups of TdT‐positive lymphomas. (A) Heatmap presentation of genetic data (see supplementary material, Figure S3 for complete mutation heatmap presentation). The mutated genes are grouped according to their association with the lymphoma entity and the SHM process. *IG::*MYC* translocations confirmed through FISH. **Genes such as *BCL2* and *MYC* that are also targets of SHM, but grouped according to their association with the disease entity. B‐ALL/LBL, B‐lymphoblastic leukaemia/lymphoma; FL, follicular lymphoma; DLBCL, diffuse large B‐cell lymphoma; HGBCL, high‐grade B‐cell lymphoma; Tr +ve, translocation‐positive; Tr −ve, translocation‐negative; IHC, immunohistochemistry; SNV, single nucleotide variant; SHM, somatic hypermutation; N/A, not available. (B) Comparison of the mutation load among the 49 SHM target genes (left panel) and all 187 genes (right panel) among DLBCL/HGBCL‐*MYC*/*BCL2*, DLBCL/HGBCL‐NOS, and B‐ALL. The red dotted line denotes the mean plus two standard deviations of mutation load from B‐ALL/LBL.

Comparative analysis among various TdT‐positive DLBCL/HGBCLs, together with B‐ALL/LBL as a reference, revealed distinct mutational features (Figure 3A and supplementary material, Figures S3, S4 and Table S4).

The TdT‐positive DLBCL/HGBCL‐*MYC*/*BCL2* group was characterised by the mutation signature associated with FL (*BCL2*, *KMT2D*, *CREBBP*, *TNFRSF14*), and those associated with high‐grade transformation (*MYC*, *TP53*, *CCND3*, *PIM1*, *B2M*, *DDX3X*, *S1PR2*). These cases also showed frequent mutations in the genes known or predicted to be targets of the somatic hypermutation (SHM) machinery, including *BCL2* and *MYC* when involved in translocation [8, 19, 20] (Figure 3A). In addition, 12 of the 13 cases of DLBCL/HGBCL‐*MYC*/*BCL2* successfully investigated showed frequent somatic mutations in their rearranged immunoglobulin heavy chain variable (*IGHV*) genes (Figure 3A and supplementary material, Table S5). These genetic changes are consistent with the GC B‐cell origin of DLBCL/HGBCL‐*MYC*/*BCL2*. In contrast, none of the B‐ALL/LBL cases showed these mutational features but displayed *TP53*, *KRAS*, and *PAX5* mutations that are frequently seen in B‐ALL/LBL [14, 15, 16] (Figure 3A). Paradoxically, one B‐ALL/LBL (Case‐28) showed a high level of SHM (86% identity to the germline) in its rearranged *IGHV* gene despite the absence of mutations in all SHM target genes investigated (Figure 3A and supplementary material, Table S5).

Among the three DLBCL‐NOS, one case (Case‐21) with *IGH*::*MYC* and *BCL6* translocation showed mutation in several SHM target genes (*MYC*, *CXCR4*, *HIST1H1C*, *HIST1H2BK*) and also evidence of SHM in its rearranged *IGHV* gene (Figure 3A). The remaining two cases were *BCL2* translocation‐positive, as shown by the presence of multiple *BCL2* mutations (Case‐20, FISH data unavailable) or interphase FISH investigation (Case‐22). Case‐20 showed the classic mutation pattern associated with FL and also mutations in several SHM target genes, while the other case (Case‐22) showed *TP53* and *NRAS* mutations but not the above mutations of transformed FL (Figure 3A). Unfortunately, it was impossible to further investigate this case due to a lack of tissue materials.

All three HGBCL‐NOS carried a *MYC* translocation and showed mutations in several SHM target genes including *MYC* (Figure 3A). One case successfully investigated also showed frequent mutations in its rearranged *IGHV* gene. These genetic data, together with CD10 expression in each case, suggest their origin from GC B cells.

Lastly, the two cases unclassifiable between B‐ALL/LBL and HGBCL‐NOS were negative for *MYC*, *BCL2*, and *BCL6* translocation, but each harboured a *TP53* mutation (Figure 3A). One case successfully investigated showed a moderate level of somatic mutations in its rearranged *IGHV* gene (supplementary material, Table S5), and this case also displayed a variant in a known SHM target gene (*CIITA*).

### Significant difference in the mutation load of SHM target genes between TdT‐positive LBCL and B‐ALL/LBL

As the number of B‐ALL/LBLs investigated in the present study was small, we compared the mutation load of a common set of SHM target genes between our TdT‐positive LBCLs (19 DLBCL/HGBCL‐*MYC*/*BCL2*, six DLBCL/HGBCL‐NOS) and a larger cohort of B‐ALL cases (*n* = 1716) from a previous study [14]. As expected, the mutation load of SHM target genes was significantly higher in DLBCL/HGBCL‐*MYC*/*BCL2* and DLBCL/HGBCL‐NOS than in B‐ALL (*p* = 2.2E−16 and *p* = 2.98E−05, respectively) (Figure 3B), with the vast majority (23/25 = 92%) of the DLBCL/HGBCL‐*MYC*/*BCL2* and DLBCL/HGBCL‐NOS harbouring a mutation load above the mean plus two standard deviations of the B‐ALL group. Similarly, the overall mutation load among the 187 genes investigated was significantly higher in DLBCL/HGBCL‐*MYC*/*BCL2* and DLBCL/HGBCL‐NOS than in B‐ALL (*p* = 2.2E−16 and *p* = 3.30E−06, respectively) (Figure 3B).

### Clonal evolution of TdT‐positive HGBCL from FL or *IGH* :: *BCL2* ‐positive premalignant B cells

Among the TdT‐positive DLBCL/HGBCLs investigated, two cases (Case‐06 and Case‐07) had metachronous lymphomas with biopsies available for comparative mutation analysis, and one further case (Case‐16) with a single microscopic nodule of an FL component, thus allowing investigation of their evolutionary history.

Case‐06 (a 45‐year‐old male) had a 1‐year history of FL and presented with malaise and a right axillary lymph node enlargement. An excision biopsy (Case‐06‐DLBCL) showed a DLBCL with a GC phenotype (CD10^+^, BCL6^+^), TdT^−^, and was negative for *MYC* translocation. The patient was treated with six cycles of O‐CHOP (obinutuzumab plus cyclophosphamide, doxorubicin, prednisolone, and vincristine) and achieved complete metabolic remission (CMR). Two years later, the patient was suspected of having a lymphoma relapse; a core biopsy of the right inguinal lymph node (not analysed by NGS due to insufficient material) and a further core biopsy of the left neck lymph node (Case‐06‐FL) a month later displayed classic FL grade 1–2, which was managed by active surveillance. Two months later, a bone marrow trephine biopsy (Case‐06‐HGBCL) revealed infiltration of medium‐ to large‐sized immature lymphoid cells that were positive for CD19, CD79a, CD43, BCL2, and TdT (moderate to strong positivity in ~80% of tumour cells), CD20 (partial) and MUM1 (partial), but negative for CD10, CD34, CD117, and BCL6. The patient was then treated with UKALL14 induction phase 1 but showed no response. Further investigations by interphase FISH revealed *IGH*::*MYC*, *IGH*::*BCL2*, and *BCL6* rearrangement, hence supporting a diagnosis of DLBCL/HGBCL‐*MYC*/*BCL2*/*BCL6*. The patient was subsequently treated with two cycles of R‐ICE (rituximab, ifosfamide, carboplatin, and etoposide), leading to a complete cytogenetic response, followed by a LACE (lomustine, cytarabine, cyclophosphamide, and etoposide)‐conditioned autologous stem cell transplant. Ten months later, the patient showed lymphocytosis (20% lymphoblasts) in peripheral blood by flow cytometry despite no evidence of lymphadenopathy by CT imaging. The patient was treated with R‐BP (rituximab, bendamustine, polatuzumab) and showed a significant clinical response, but died a month later.

**Figure 4:**
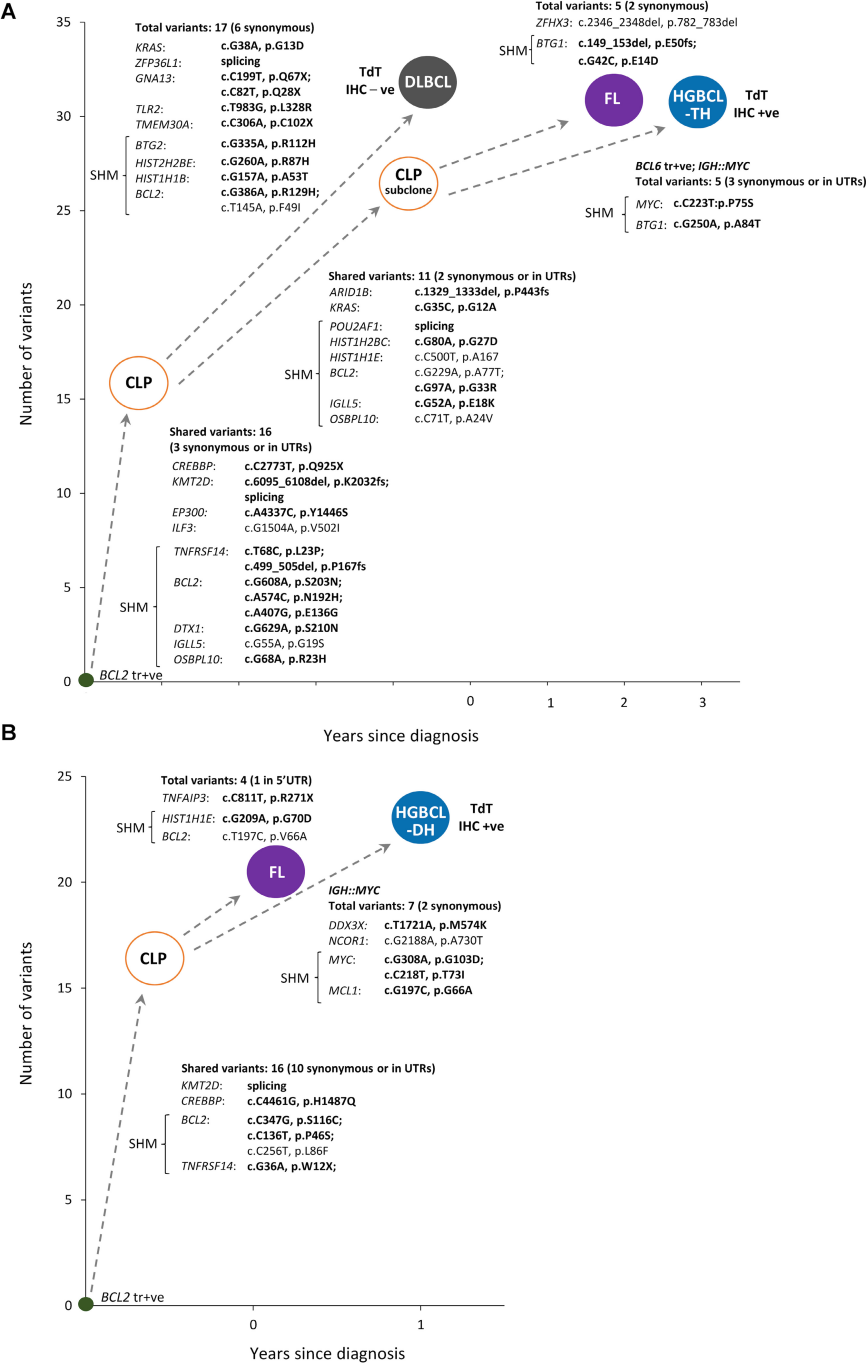
Clonal evolution of TdT‐positive HGBCL as revealed by mutation profiling. The number of all clonal variants [including pathogenic (in bold), benign, synonymous variants and those in UTR regions] that occurred at each stage of lymphoma development are provided, and only nonsynonymous variants are presented in detail. The shared and distinct clonal variants in paired lesions and their predicted evolutionary trajectory in each case are illustrated. (A). Case‐06 shows divergent evolution of DLBCL, FL, and TdT‐positive HGBCL‐TH (*BCL2*/*MYC*/*BCL6* translocation) from a common CLP cell population, with the latter two lesions originating from a CLP subclone. (B). Case‐07 displays divergent evolution of FL and TdT‐positive HGBCL‐DH (*BCL2*/*MYC* translocation) from a common CLP cell population.

Targeted NGS analysis revealed both common and distinct variants among the DLBCLs, FLs, and HGBCL‐*MYC*/*BCL2*/*BCL6* (Figure 4A). These metachronous lymphomas shared 16 common clonal variants including 11 potentially pathogenic and five benign/synonymous/UTR changes, hence confirming their clonal relationship. The DLBCLs had 17 private clonal variants including ten potentially pathogenic changes, while the FLs and HGBCL‐*MYC*/*BCL2*/*BCL6* harboured different private variants. Intriguingly, the FLs and HGBCL‐*MYC*/*BCL2*/*BCL6* had both common (six potentially pathogenic and five benign/synonymous/UTR changes) and unique variants (five variants in FLs including two potentially pathogenic changes; five variants in HGBCL‐*MYC*/*BCL2*/*BCL6* including two potentially pathogenic changes). These mutation patterns indicated divergent evolution of these different lymphomas from a common lymphoma precursor (CLP) cell population, with the FLs and HGBCL‐*MYC*/*BCL2*/*BCL6* derived from an intermediate subclone of the CLP cell population (Figure 4A).

Case‐07 (a 42‐year‐old male) was a referral; a lymph node excision biopsy (Case‐07‐FL) showed an FL grade 2, which was BCL2^+^, CD10^+^, but TdT^−^. The patient was treated with three cycles of R‐CHOP and then switched to two cycles of R‐ICE due to disease progression, but showed no response. A further biopsy of an inguinal lymph node 4 months later showed a high‐grade transformation with the large cell component being CD20^−^, BCL2^+^, CD10^+^ (weak), BCL6^−^, MUM1^+^, Ki67^+^ (100%), TdT^−^, and TP53^+^. The patient was treated with one cycle of R‐DHAP (rituximab–dexamethasone, cytarabine, cisplatin) and venetoclax, and then switched to R‐GemOx (rituximab, gemcitabine, and oxaliplatin) + Polivy (polatuzumab vedotin) due to disease progression. Subsequently, the patient underwent abdominal exploration due to suspected splenic rupture, which instead revealed a peri‐gastric tumour mass. A biopsy of the tumour mass (Case‐07‐HGBCL) showed a transformed lymphoma with the large cell component being BCL2^+^ (50%), TdT^+^ (60%), MYC^+^ (80%). Interphase FISH showed both *IGH*::*MYC* and *BCL2* translocation, thus supporting a diagnosis of HGBCL‐*MYC*/*BCL2*. The patient died 1 week later after diagnosis. Targeted sequencing analysis of the initial FL and peri‐gastric HGBCL‐*MYC*/*BCL2*‐DH revealed 16 common clonal variants (five potentially pathogenic and 11 benign/synonymous/UTR changes), and also four and seven private variants, respectively. These, together with identical *IGH* rearrangements between the two lymphomas, indicated their divergent evolution from a CLP cell population (Figure 4B).

**Figure 5:**
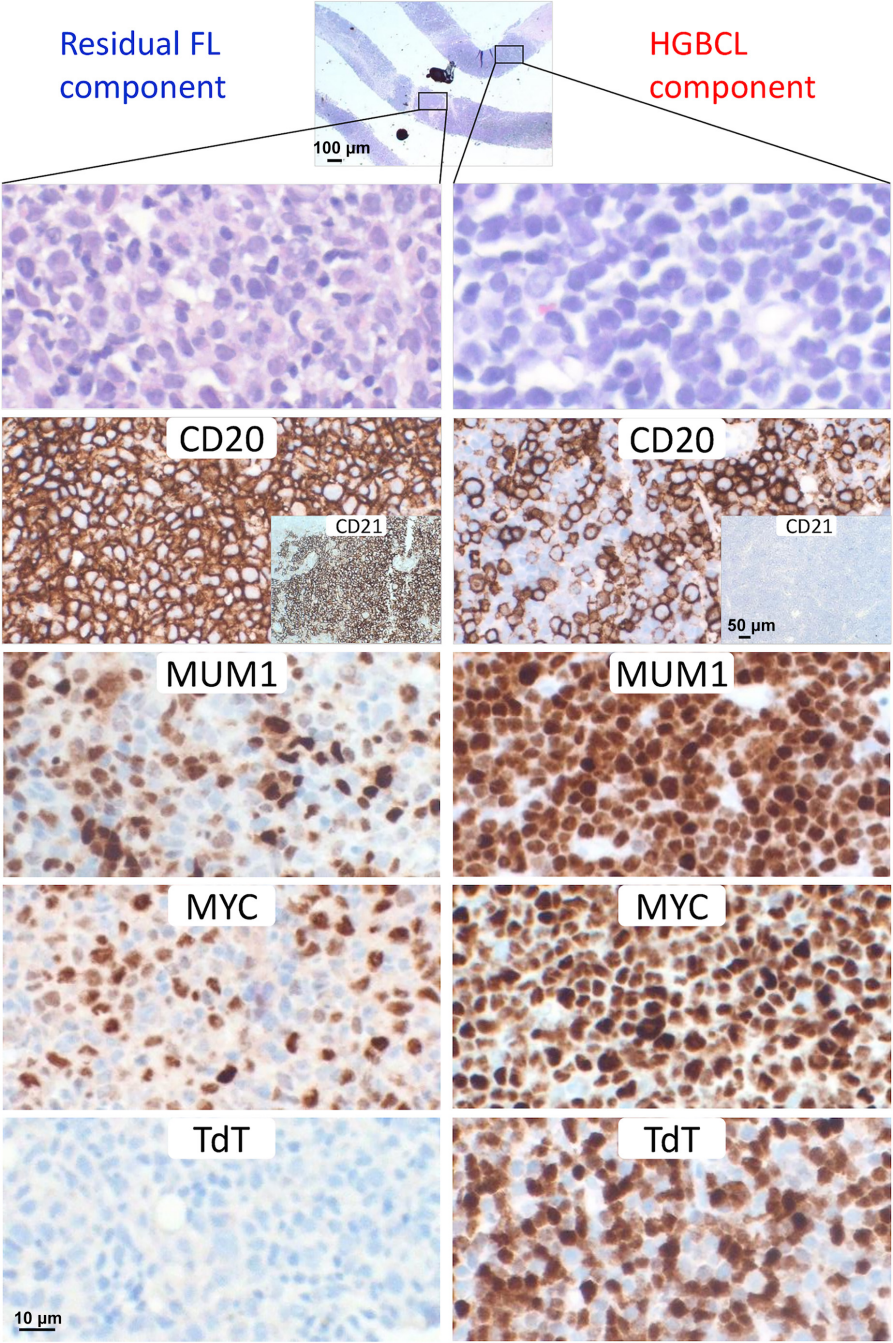
Histological and immunophenotypic presentation of Case‐16 with HGBCL‐*MYC*/*BCL2* and a single microscopic nodule of the follicular lymphoma (FL) component. The HGBCL cells show diffuse PAX5 positivity (not shown), but CD20 positivity in ~50% of tumour cells, and are positive for MUM1, MYC (100%), and TdT (~40%), while the FL component shows scattered positivity for MUM1 (~30%) and MYC (~40%), but negative TdT expression.

Case‐16 (a 77‐year‐old male) presented with left leg swelling with multiple enlarged lymph nodes in the left groin. Multiple core biopsies of the lymph nodes showed HGBCL with a single microscopic nodule of an FL component. The HGBCL cells were positive for PAX5, CD20 (~50%), CD10, BCL2, MUM1, MYC (100%), and TdT (~40%), but negative for CD34 and BCL6, while the FL cells were diffusely positive for PAX5, CD20, CD10, BCL2, and BCL6, partially expressed MUM1 (~30%) and MYC (~40%), but were negative for TdT and CD34 (Figure 5). Interphase FISH showed both *MYC* and *BCL2* translocation in the HGBCL and FL, with *MYC* translocation in the FL component only seen in scattered large nuclei. Clonal analysis of the rearranged *IG* genes based on microdissected HGBCL and FL components showed identically sized *IGH* and *IGK* products (supplementary material, Figure S5), thus confirming their clonal relationship. Together, these findings suggest that the HGBCL originated from the FL following acquisition of a *MYC* translocation. In line with this speculation, the HGBCL harboured characteristic mutations associated with FL, high‐grade transformation, and SHM activities (Figure 3A).

### Aberrant TdT expression and *MYC* translocation

Since the majority of TdT‐positive DLBCL/HGBCLs originate from *IGH*::*BCL2*‐positive FL or its precursor cell population following acquisition of a *MYC* translocation, this raises the question of whether the aberrant TdT expression might be due to dysregulated transcriptional activity of highly expressed MYC. To investigate this and identify appropriate cell lines for further *in vitro* studies, we examined TdT expression in ten DLBCLs (including five cell lines with *MYC/BCL2‐*DH and an additional line with *MYC* translocation) and one Burkitt lymphoma cell line by immunocytochemistry. None of these cell lines showed any positive TdT staining. Additionally, an extensive bioinformatics search did not reveal any evidence that TdT might be a potential MYC transcriptional target.

## Discussion

The differential diagnosis of TdT‐positive DLBCL/HGBCL and B‐ALL/LBL may pose profound problems due to the overlapping features of these two diagnostic families/entities especially when *MYC* rearrangements are encountered in a CD34‐negative aggressive B‐cell neoplasm in an adolescent or a young adult patient. By investigation of a spectrum of TdT‐positive B‐cell lymphomas including DLBCL/HGBCL‐*MYC*/*BCL2*, DLBCL‐NOS, HGBCL‐NOS, B‐ALL/LBLs, and cases unclassified, the present study has highlighted several points that would aid the differential diagnosis between TdT‐positive DLBCL/HGBCL and B‐ALL/LBL.

Loss of expression of pan B‐cell antigens, such as CD20, has been used to argue for a diagnosis of B‐ALL/LBL, while the expression of mature B‐cell antigens such as BCL6 and MUM1 favours the diagnosis of mature rather than precursor B‐cell lymphoma. However, none of these immunophenotypic features is specific enough for reliable differential diagnosis between TdT‐positive LBCL/HGBCL and B‐ALL/LBL. Our data clearly demonstrate that a high proportion (15/23 = 65%) of TdT‐positive LBCL/HGBCLs show complete or partial loss of CD20 expression, while MUM1 can also be positive in B‐ALL/LBL [1]. Nonetheless, CD34 is consistently negative in TdT‐positive LBCL, but a lack of CD34 expression can occur in a high proportion of B‐ALL/LBLs, as shown in the present and previous study [21, 22, 23, 24].

Apart from the detection of chromosomal translocations, mutation analysis is highly valuable in the differential diagnosis between TdT‐positive LBCL and B‐ALL/LBL. Like conventional DLBCL/HGBCL‐*MYC*/*BCL2*, the TdT‐positive cases with *MYC*/*BCL2* translocation harboured a characteristic mutation profile, including mutation signatures associated with FL and also its high‐grade transformation [8, 9, 10, 11, 12]. TdT‐positive DLBCL‐NOS and HGBCL‐NOS also showed frequent mutations commonly seen in these entities, albeit less striking as those of DLBCL/HGBCL‐*MYC*/*BCL2*. Furthermore, except for one case (Case‐22), all the other DLBCL‐NOS and HGBCL‐NOS exhibited frequent mutations in the SHM target genes, along with CD10 expression, indicating their origin from GC B cells. This was also supported by findings of frequent mutations in their rearranged *IGHV* genes in each case successfully investigated. In contrast, B‐ALL/LBL was largely driven by chromosome translocations and copy number changes, harbouring few mutations [14, 15, 16], and particularly lacking somatic variant‐associated SHM activities as they originate from precursor B cells that have not yet undergone antigen affinity maturation in the peripheral lymphoid tissues [13].

As targeted sequencing by NGS is increasingly used in the diagnosis and sub‐classification of B‐cell lymphoma, it is important to include genes targeted by the SHM machinery and document their somatic changes as discussed above. It is necessary to report all somatic variants identified including both synonymous and nonsynonymous variants, as well as those present in the 5' UTR and intronic regions to maximise their utility as biomarkers in delineating the dichotomy of cell of origin between pre‐GC and GC/post‐GC B cells. This study serves as a proof of principle. Future studies are required to standardise the list of genes and their genomic regions to be covered by NGS, and also to establish the optimal method for quantifying somatic variants and their threshold value for reliable prediction of lymphoma cell‐of‐origin.

Of the two cases with diagnostic ambiguity between B‐ALL/LBL and HGBCL‐NOS, the genetic data obtained failed to provide any strong evidence for their definitive diagnosis. Apart from *TP53* mutation, both cases lacked the other mutations seen in HGBCL‐NOS. Case‐27 showed a single mutation in an SHM target gene (*CIITA*) and a moderate level (95%) of SHM in its rearranged *IGHV*, while the other case showed no mutation in the SHM target genes investigated. Such a low level of somatic mutations in the rearranged *IGHV* genes has been reported in B‐ALL previously [25]. These mutations, together with their variable expression of pan‐B‐cell (CD20) and mature B‐cell (BCL6, MUM1) markers, may argue for the existence of grey zone cases between B‐ALL/LBL and HGBCL‐NOS.

The mechanism for aberrant TdT expression in LBCL is unclear. Given that aberrant TdT expression is often associated with partial or complete loss of expression of pan‐B cell antigen (CD20), it is possible that these cases develop a defect in the transcriptional network that controls the B‐cell programme. In line with this speculation, there is no evidence of loss of CD20 expression in conventional DLBCL/HGBCL, and this is further reinforced by a retrospective review of 15 TdT‐negative and *MYC* translocation‐positive DLBCLs/HGBCLs. Among the transcription factors, *PAX5* genetic changes including the mutation p.P80R are frequently seen in B‐ALL/LBL, and these genetic changes are thought to affect B‐cell programming, thereby contributing to leukaemia development [26, 27]. *PAX5* is a SHM target and multiple mutations were observed in a case of HGBCL‐*MYC*/*BCL2*‐DH (Case‐08), affecting the paired box domain and also the highly conserved octapeptide motif (Figure 3A and supplementary material, Figure S4). In this context, it is worth noting mutations in several other transcription factors that regulate early B‐cell development, such as *ETS1* (SHM target), *FOXO1* (SHM target), *TCF3* (encoding E2A), and *POU2F2* (encoding OCT2) [28] (Figure 3A and supplementary material, Figure S4). It remains to be investigated whether mutations in these transcription factors and related regulators may dysregulate the B‐cell programme, causing aberrant TdT expression and also perturbing B‐cell antigen expression.

In conclusion, our findings demonstrate that mutation profile analysis, including SHM target genes, is highly valuable in the differential diagnosis between TdT‐positive DLBCL/HGBCL and B‐ALL/LBL.

## Author contributions statement

M‐MT, FC, ZC, EM, JM, AES, FG and M‐QD performed FISH, targeted NGS, data collection and analyses. LR‐B, AW, KSK, CE, ES, LH, LK, JTG, CL, LX, LM, LP, LMRG, NL‐H, TG, JK, WK, IO, AR and GO were responsible for case contribution and pathology. M‐QD, LR‐B and GO conceived the study, and were responsible for study coordination and research funding. M‐QD and M‐MT wrote and prepared the manuscript, with contributions from all authors. All authors read and approved the final manuscript.

## Supporting information


Supplementary materials and methods
**Figure S1**. Sequencing coverage of targeted NGS
**Figure S2**. Histological and immunophenotypic presentation of Case‐27 unclassified between HGBCL‐NOS and B‐ALL/LBL
**Figure S3**. Heatmap presentation of genetic data
**Figure S4**. Mutations identified in genes encoding for transcription factors that are critical for early B‐cell development
**Figure S5**. Comparative analysis of the high‐grade and follicular lymphoma components in Case‐16

**Table S1.** Antibodies used for immunohistochemistry
**Table S2**. List of the genes investigated by TWIST capture and NGS
**Table S3**. Primers used for immunoglobulin heavy chain (*IGH*) sequencing
**Table S4**. Variants detected by 187‐gene panel sequencing
**Table S5**. Immunoglobulin heavy chain (*IGH*) usage detected using adapted BIOMED‐2 primers coupled with NGS

## Data Availability

All core data generated or analysed during this study are included in this published article, and additional raw data are available from the corresponding author on reasonable request.
