# Supplementary material for: Mutation profiling in differential diagnosis between TdT‐positive high‐grade/large B‐cell lymphoma and B‐lymphoblastic leukaemia/lymphoma
Source: J Pathol. 2025 Oct 6;267(4):410–23. doi: 10.1002/path.6476 (PMC12596909; doi:10.1002/path.6476)
Supplement: Supplementary file 1 — Supplementary materials and methods Figure S1. Sequencing coverage of targeted NGS Figure S2. Histological and immunophenotypic presentation of Case‐27 unclassified between HGBCL‐NOS and B‐ALL/LBL Figure S3. Heatmap presentation of genetic data Figure S4. Mutations identified in genes encoding for transcription factors that are critical for early B‐cell development Figure S5. Comparative analysis of the high‐grade and follicular lymphoma components in Case‐16 [file PATH-267-410-s002.docx]

**Mutation profiling in differential diagnosis between TdT-positive high-grade/large B-cell lymphoma and B-lymphoblastic leukaemia/lymphoma**

M-M Tzioni *et al. J Pathol* <https://doi.org/10.1002/path.6476>

**Supplementary materials and methods**

**Supplementary Figures S1–S5**

**Supplementary Tables S1–S5 (provided in a separate Excel file)**

**Supplementary materials and methods**

*PCR and next-generation sequencing of the rearranged immunoglobulin heavy chain* (IGH) *genes*

The library preparation was carried out using a two-step PCR protocol. Modified BIOMED-2 FR1-JH or FR2-JH primers were utilised in the first PCR to amplify the rearranged *IGH* genes (supplementary material, Table S3). PCRs were performed using the FastStart High Fidelity PCR System (Roche, Basel, Switzerland). PCR reactions were undertaken in a 25-μl volume that contained the following master-mix components with the specified final concentration: 4.5 mm MgCl_2_, 0.05 U/μl FastStart High Fidelity Polymerase Enzyme, 5% DMSO, 200 μm of PCR-grade nucleotide mix, 1× FastStart High Fidelity Buffer (without MgCl_2_), with a final primer concentration of 0.16 μm. Each reaction was performed in duplicate, using 25 and 50 ng of genomic DNA, respectively. The following PCR conditions were used: 95 °C for 10 min, 40 cycles of 96 °C for 45 s, 60 °C for 45 s, and 72 °C for 90 s, and the final extension, 72 °C for 10 min and 25 °C for 10 min.

Successfully amplified clonal PCR products were purified using SPRIselect Beads (Beckman Coulter, Brea, CA, USA) following the manufacturer’s protocol, with a bead-to-PCR product ratio of 0.8:1. A second PCR was performed to anneal sequencing adapters and unique dual indexes (supplementary material, Table S3) (SureSelect XT Low Input Indexes; Agilent Technologies, Santa Clara, CA, USA) to the purified PCR products, using the FastStart High Fidelity PCR System (Roche) under the same reaction conditions as the initial amplification, with a primer concentration of 4 μm. For each reaction, 5 ng of the purified PCR product from each sample was used. The following PCR conditions were used: 95 °C for 10 min, ten cycles of 95 °C for 15 s, 60 °C for 30 s, and 72 °C for 60 s, and the final extension, 72 °C for 3 min. Barcoded PCR products were pooled and sequenced using the Illumina NovaSeq X platform (Illumina, San Diego, CA, USA) (paired-end 250 bp protocol).


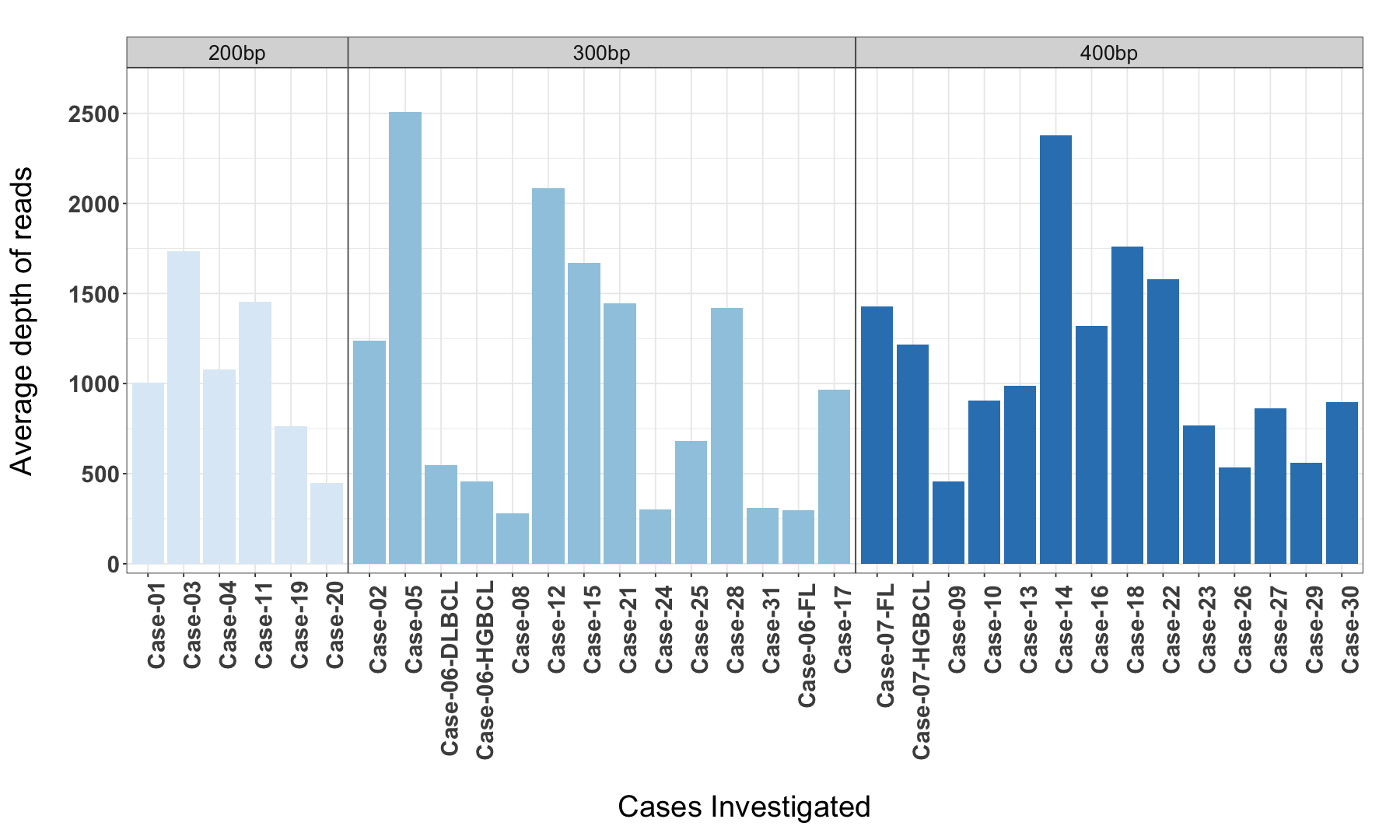


**A**

**B**


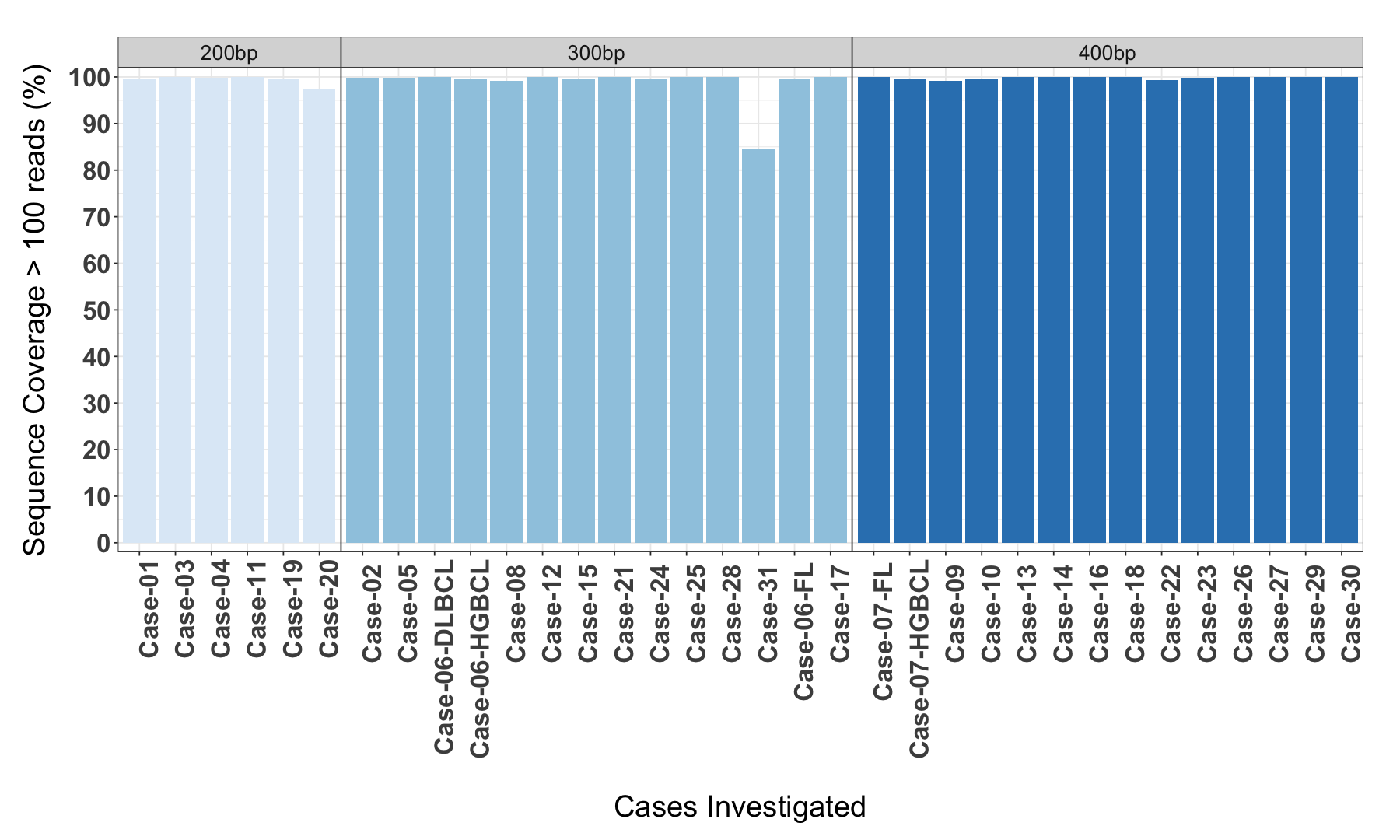


*

*

*

*

*

*

*

*

*

*

*

*

*

*

*

*

*

*

**Figure S1.** **Sequencing coverage of targeted NGS.** DNA quality as measured by PCR of variably sized genomic fragments is indicated at the top of each panel. *Samples subjected to targeted NGS in duplicates.

**Figure S2. Histological and immunophenotypic presentation of Case-27 unclassified between HGBCL-NOS and B-ALL/LBL.** The bone marrow trephine biopsy shows sheets of lymphoblastoid cells that display diffuse PAX5 but partial CD20 expression. The lymphoma cells are positive for CD10, BCL6, MUM1 (weak), MYC, and TdT (~30%), but negative for CD34.


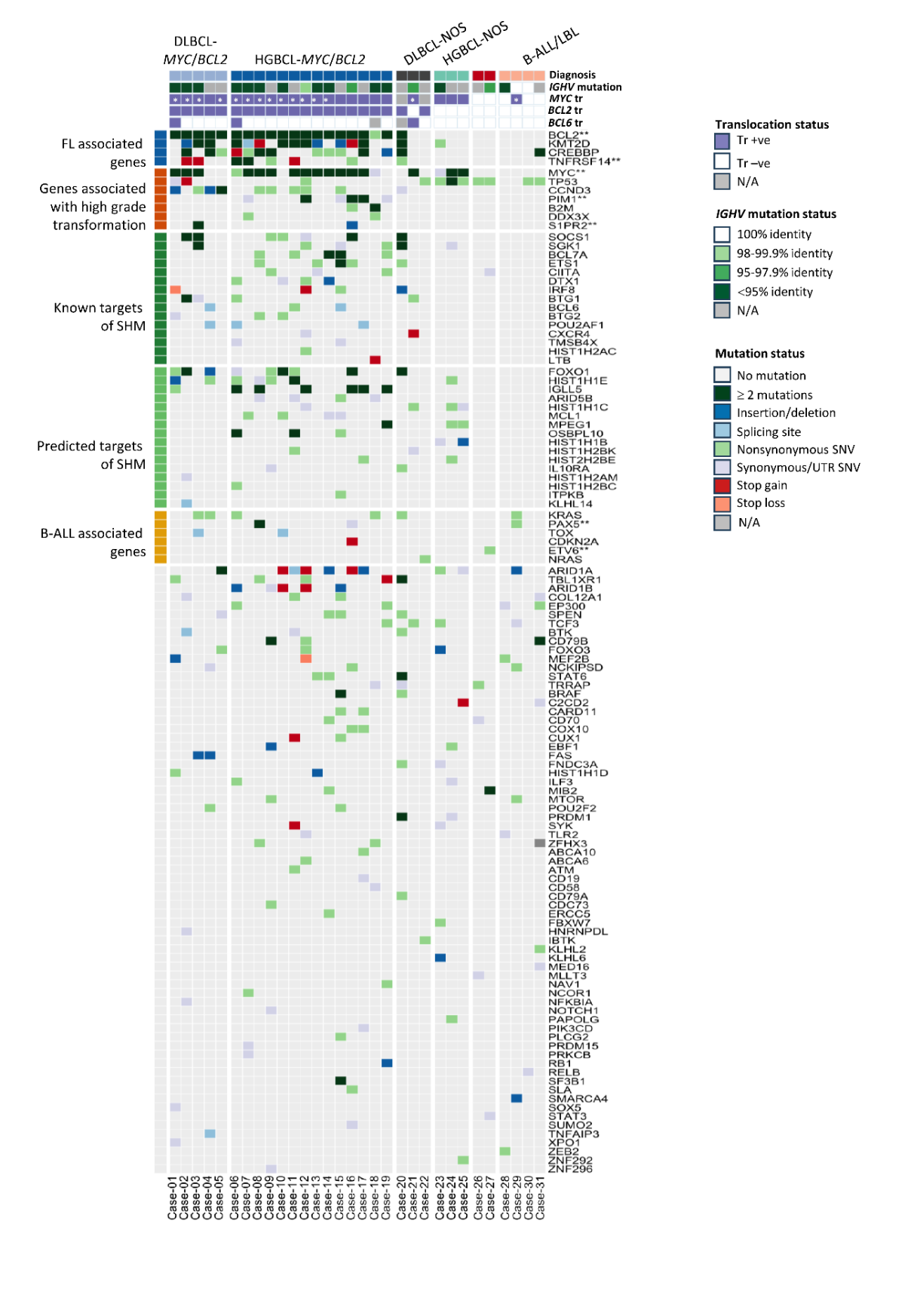


**Figure S3. Heatmap presentation of genetic data.** The mutated genes are grouped according to their association with the lymphoma entity and the SHM process. **IG*::*MYC* translocations confirmed through FISH. **Genes such as *BCL2* and *MYC* that are also targets of SHM, but grouped according to their association with the disease entity. B-ALL/LBL, B-lymphoblastic leukaemia/lymphoma; FL, follicular lymphoma; DLBCL, diffuse large B-cell lymphoma; HGBCL, high-grade B-cell lymphoma; Tr +ve, translocation-positive; Tr −ve, translocation-negative; IHC, immunohistochemistry; N/A, not available; SNV, single nucleotide variant; SHM, somatic hypermutation.


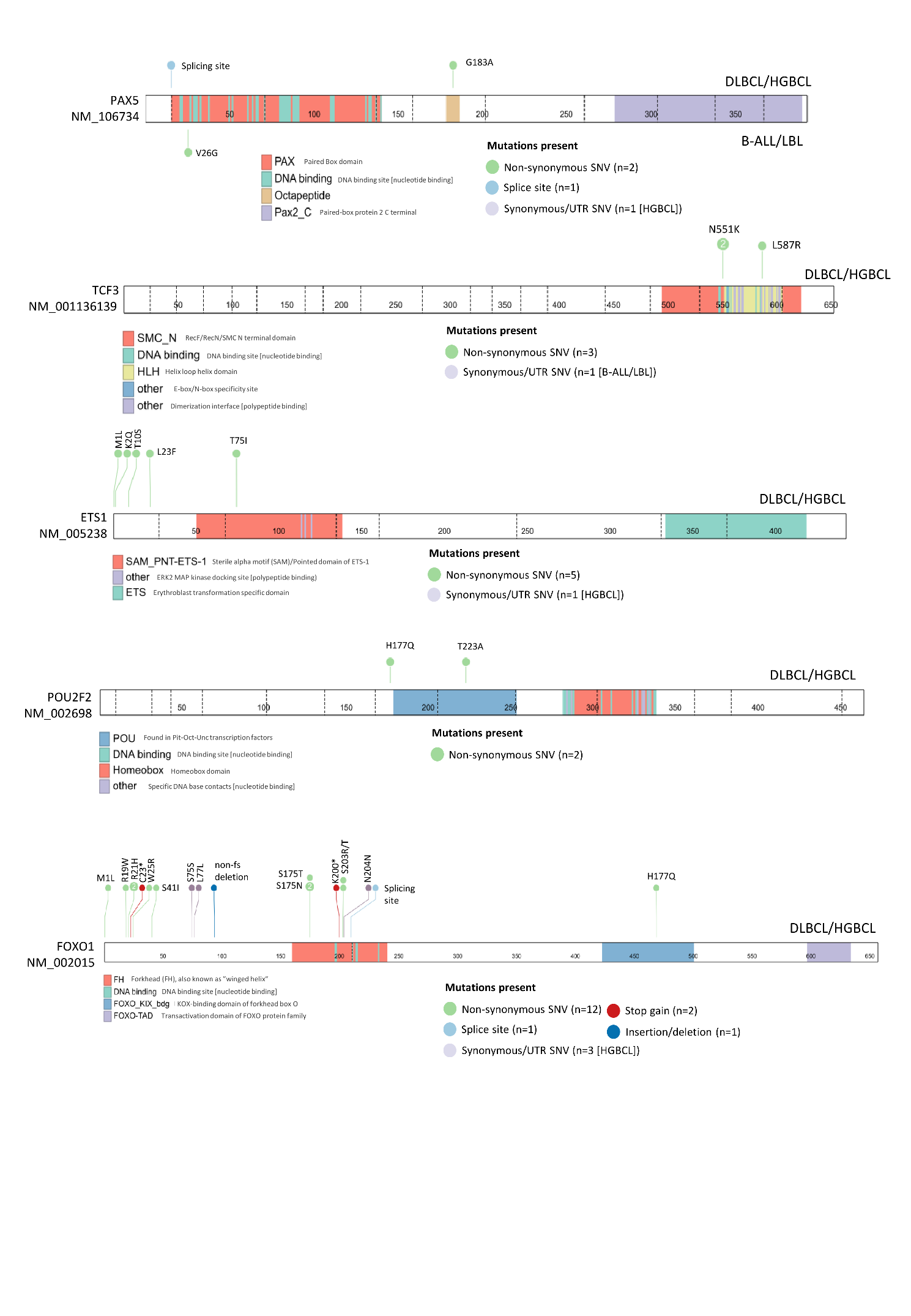


**Figure S4.** Mutations identified in genes encoding for transcription factors that are critical for early B-cell development.


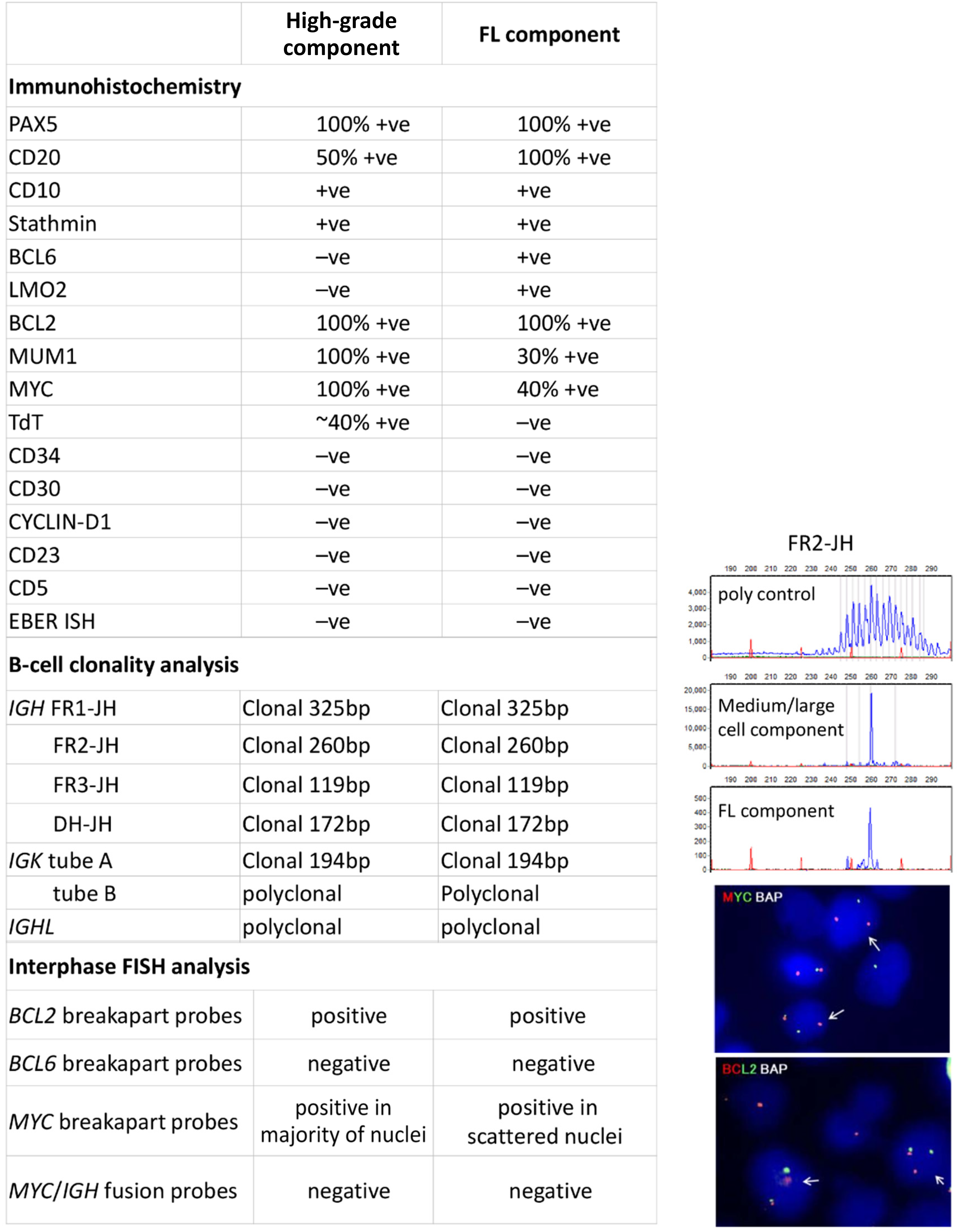


**Figure S5.** Comparative analysis of the high-grade and follicular lymphoma components in Case-16. BAP, breakapart probe.
